# The North-Western Fever Hospital

**Published:** 1899-08-12

**Authors:** 


### The North-Western Fever Hospital.

The annual report of the Metropolitan Asylums Board is an interesting document, showing, as it does, how much is done for the inhabitants of London when they have the misfortune to be attacked with infectious disease. At the same time, it has to be remembered that, dealing, as it does, with large numbers, most of its conclusions are expressed as averages, which, useful as they are from a statistical point of view, are far from expressing what happens to any particular patient or even in any particular hospital; and what we propose to do in the following remarks is to show what extreme differences lie buried in these averages, and to ask—although, we fear, not to answer—the question, What explanation is to be offered as to the wide difference which exists between the numbers of the medical and of the nursing staffs, the character of the treatment, and the results, as expressed by the mortality, at the different hospitals? A table is given in the report showing the average daily number of patients under treatment, and the average daily number of staff employed, at each of the fever hospitals during the year 1898. The same table also shows the proportions of the nursing staff and of the total staff to the patients in each case, and data are given from which, by a simple calculation, the same proportion can be worked out in regard to the medical staff. It is stated that "at the hospitals for acute cases the proportion varied from one nurse to 3·5 at the Eastern Hospital and 3·5 at the North-Western Hospital to one nurse to 2·6 at the Park Hospital, and the total staff at the two former hospitals was as one to 1·5 and 1·0 patients respectively, and at the latter hospital as one to 1·3." Here, then, we come across the curious fact that the hospital in which the proportion of total staff to patients is the highest—so high, indeed, that the staff and the patients are equal—the proportion of nurses to patients is the lowest, namely, one nurse to 3·5 patients. On looking at the table we also find that at this same hospital the proportion of doctors to patients is also far lower than is the case at any other of the hospitals for acute diseases, there being, in fact, only one medical officer to 92 patients, while at the South-Western Hospital there is one medical officer to every 49 patients. These two hospitals may indeed be usefully compared in other respects. They are alike in this—that they are the two most highly staffed hospitals under the management of the Board. Here, however, the likeness ceases, for while the South-Western Hospital has the largest proportion of medical officers, and next but one to the largest proportion of nurses, the North-Western has the lowest proportion of medical officers and the lowest proportion of nurses. Now let us return to results. The average death-rate from scarlet fever at all the hospitals of the Board is 4·12 per cent. The very highest death-rate from this disease is at the Western Hospital, which again is the hospital which stands next to the North-Western in regard to the number of patients to each doctor and to each nurse. The scarlet fever death-rate at this hospital is 5·75 per cent., but next to it comes the death-rate at the North-Western, namely, 5·25 per cent. The lowest death-rate of all is found at the South-Western, a hospital with a full medical and nursing staff, which has a mortality of only 2·16 per cent., or considerably less than half that of its North-Western rival. Now take diphtheria. The average mortality at all the hospitals is 15·37 per cent.; at the North-Western it is the highest of all, namely, 19·87 per cent.; at the South-Western it is 11·96 per cent. Then take enteric fever, the average mortality of which at all the hospitals is 17·73. At the North-Western it is 22·84 per cent.

What is clear is that the North-Western Hospital has a mortality in each of the disease which it treats far above the average, and, except in the case of scarlet fever, far above that of any other hospital. But searching about for some explanation of this curious state of affairs we come across other facts which, however little they may have to do with the matter, are at least of considerable interest. The first of these is as to the use of antitoxin. It appears that this is used in a far smaller proportion of the cases admitted at the North-Western Hospital than at any other hospital under the Board. Then we find in a table showing the incidence of complications in cases of enteric fever that hæmorrhage, perforation, and peritonitis all occur in a larger proportion of the cases at the North-Western than at the other hospitals, and although this might no doubt be put forward as indicating that the cases sent there are more severe than usual, it may of course bear quite another interpretation. Moreover, we are not aware of any process of selection by which the more severe cases find themselves at Hampstead.

The outcome of the whole thing is this: the North-Western Hospital seems to be distinguished by the largeness of its general staff; the smallness of its medical and nursing staffs; by its heavy mortality, not in regard to one disease alone but to all which are taken in, both scarlet fever, diphtheria, and enteric fever; by the comparative infrequency with which the most modern treatment of diphtheria is made use of; and by the frequency of certain complications of enteric fever, the occurrence of which it would not be improper to take as a test of the character of the nursing. We think that the whole subject is one which urgently calls for an independent inquiry.

